# Re-discovering NK cell allo-reactivity in the therapy of solid tumors

**DOI:** 10.1186/s40425-016-0159-4

**Published:** 2016-09-20

**Authors:** Luca Castagna, Domenico Mavilio

**Affiliations:** 1 Hematology and Bone Marrow, Transplant Unit, Humanitas Cancer Center, Rozzano, Milan Italy; 2 Department of Medical Biotechnologies and Translational Medicine, University of Milan, Milan, Italy; 3 Unit of Clinical and Experimental Immunology, Humanitas Clinical and Research Center, Rozzano, Milan Italy

**Keywords:** NK cells, Allo-reactivity, Adoptive cell therapies, Solid tumors, Lymphomas

Currently, refractory lymphoma or advanced solid tumors have an unfavorable prognosis as conventional therapies are often not curative while providing high levels of toxicity. Indeed, the overall survival of these patients is relatively short with a poor quality of life. This clinical evidence generates a relevant unmet medical need. In the last few years, a renewed interest on adoptive cellular therapies emerged in the field of tumor-immunology due to the remarkable advances in regard to our knowledge of the mechanisms employed by immune system to fight cancer counterbalanced by the pathogenic loops deployed by tumors to escape immune responses. All these gained insights on cancer physiopathology greatly pushed the development of novel technologies in biomedical field that, in turn, led to the discovery of new categories of drugs and alternative therapeutic approaches. Indeed, the use of check-point inhibitors, T cells with engineered chimeric antigen receptors (CARs) and bi-specific T cell engagers (BiTE) showed impressive therapeutic potentials in the cure of advanced tumors and are currently being included in the in the armamentarium of clinicians [1].

Among the different approaches of anti-tumor adoptive cell transfer, allogeneic stem cell transplantation (allo-SCT) represents the first and most consolidated form of immunotherapy on the basis of its graft versus tumor (GvT) effect. In this context, immune cell allo-reactivity can be either associated with antigen presentation exerted by class-I major histocompatibility complex (MHC-I) as in the case of CD8 and CD4 T cells or, alternatively, is antigen-independent as occurs with NK cells. However, GvT is counterbalanced by important toxicity and non-relapse mortality [2]. Many attempts have been performed, outside the allo-SCT contest, to exploit the allo-reactivity activities of immune cells in order to limit or bypass the toxicity experienced with allo-SCT. In the early 80s’ donor lymphocyte infusions (DLI) with allo-activated haplo-identical cells from family members were infused in patients affected by hematological diseases or solid tumors. These study demonstrated that a temporary mixed chimerism and tumor reduction often associated to graft versus host disease (GVHD) is possible [3, 4]. It has been also reported that, after standard chemotherapy, the DLI with G-CSF-primed immune cells from related haplo-identical donors in aged patients affected by acute myeloid leukemia can significantly reduces the rate of tumor relapse [5].

NK cell recognition of “self” to ensure immunologic tolerance versus autologous targets does not require a prior sensitization and relies on large family of inhibitory NK cell receptors (iNKRs) including Killer cell immunoglobulin-like receptors (KIRs) and C-type lectins that recognize specific alleles of MHC-I. The lack of self-MHC-I on tumor-transformed cells makes them susceptible to NK cell mediated lysis via the engagement of several activating NK cell receptors (aNKRs) that bind their ligands expressed on cancer cells (i.e. missing self hypothesis) [6, 7]. Several protocols of adjuvant therapies based on NK cell allo-reactivity have been extensively used in clinics for both hematologic and non-hematologic malignancies either alone or in combination with antibodies and tumor-sensitizing drugs. Indeed, adoptive cell transfer of allogeneic NK cells have been used for the treatment of leukemia, colorectal cancer, hepatocellular cancer, lymphoma and melanoma. The major risk with these procedures is the development of GVHD and several precautions have been put in place to avoid this unfavorable clinical event, such as the infusion of CD3 depleted high pure NK cells or the selection of HLA matched donors [8]. Important insights into NK cell function in cancer eradication originated from the knowledge gained from mismatched NK cells in allo-SCT setting, where these innate immune effectors mediate a remarkable graft-versus-leukemia (GvL) effect towards tumor cells of recipient without attacking their normal tissues, thus limiting the onset of GVHD. Moreover, it has been demonstrated HLA-mismatched NK cells in haploidentical allo-SCT improve the overall survival of patients affected by acute myeloid leukemia by controlling tumor relapse without causing GVHD. The reduced priming of alloreactive donor T cells as a consequence of NK cell killing of recipient antigen-presenting cells and/or of a direct inhibition of activated allo-reactive T cells have been postulated as explanation of the low frequency of GVHD [9–13]. However, a recent study reported that the infusion of allogeneic IL-15 plus 4-1BBL activated human NK cell-based DLI induced acute GVHD in five out of nine patients affected by solid tumors [14]. Finally, several other variables hampering NK cell allo-reactivity against cancer should be considered. Among these, there are the ability of NK cell to infiltrate the tumor mass via the expression of homing receptors (i.e. chemokines or others) as well as the capacity of cancer cells to impair NK cell effector-functions or to became resistant to NK cell lysis (i.e. induction of inhibitory immunologic pathways or lack of expression of aNKR ligands on their surface).

In the paper published by Yang Y. et al. [15] in this issue of Cancer Immunology research, NK cells from random and fully mismatched donors were first selected and expanded to be then infused in patients with either solid tumors (*n* = 18) or lymphomas patients (*n* = 2) in the context of a phase I clinical trial. After testing escalated doses of administered NK cells, authors did not report a maximum tolerated dose (MTD) as only grade 1 and 2 toxicity was observed in the absence of GVHD. The study also shows that the “safe” dose of adoptively transferred NK cells that can be infused up to 3 times at the dose of 3 x 10^7^ cells/kg/week. Another important point addressed by this work is the persistence of fully mismatched NK cells in the recipients associated to their anti-tumor effector-functions. Allo-reactive NK cells were detected in the peripheral blood of recipients up to 4 days after infusion with only 8 patients that achieved disease stabilization while 9 progressed. Based on these data, it can be drawn that the lifespan of mismatched NK cells is quite short as they are quickly rejected by recipient immune system. On the other hand, considering the all patients were affected by advanced solid tumors, disease stabilization can be considered an encouraging result likely associated with the infusion of allo-reactive NK cells endowed with great anti-tumor potential. Moreover, as also previously reported [11], Yang Y. et al. postulate that the simultaneous administration of immunosuppressive drugs can prolong the persistence of infused cells and boost the efficacy. Finally, the choice of random healthy donors represents another important information provided by this clinical trial to, at least try, resolve the problem of finding a reliable and effective source of alloreactive NK cells. As reported in haploidentical clinical trials of allo-SCT [10], this strategy is aimed to develop a quick and “low cost/low-tech” process to generate clinical grade anti-cancer NK cells. Indeed, this phase I trial confirm that NK cells from random donors are safe without major toxic effects, thus advocating for larger studies to confirm their clinical efficacy. The exposure to cytokines, viral antigens and haptens is associated with both modulation of NK cell effector function and with the generation of a newly disclosed subset of CD57^pos^/NKG2C^pos^/KIR^pos^ NK cells endowed with adaptive traits [16, 17]. Hence, further investigation are required to better determine the impact of these “memory-like” NK cells in allo-reactive response against cancer cells occurring in a malignant microenvironment enriched with several immunogenic stimuli such as viruses or cancer-induced haptens or inflammatory soluble factors.

## Related article

Phase I study of random healthy donor-derived allogeneic natural killer cell therapy in patients with malignant lymphoma or advanced solid tumors by Yaewon Yang, Okjae Lim, Tae Min Kim, Yong-Oon Ahn, Hana Choi, Hyejin Chung, Bokyung Min, Jung Hyun Her, Sung Yoo Cho, Bhumsuk Keam, Se-Hoon Lee, Dong-Wan Kim, Yu Kyeong Hwang, and Dae Seog Heo.
